# Theory of professional bonds: description and theoretical analysis based on the Meleis’ model with Delphi strategy^*^

**DOI:** 10.1590/1980-220X-REEUSP-2022-0054en

**Published:** 2022-06-03

**Authors:** Manuela Gomes Campos Borel, Maira Buss Thofehrn, Cristina Arreguy-Sena, Romanda da Costa Pereira Barboza Lemos, Candida Caniçali Primo, Marcos Antônio Gomes Brandão

**Affiliations:** 1 Universidade Federal do Rio de Janeiro, Escola de Enfermagem Anna Nery, Programa de Pós-graduação Stricto Sensu em Enfermagem, Rio de Janeiro, RJ, Brazil.; 2 Universidade Federal de Pelotas, Faculdade de Enfermagem, Pelotas, RS, Brazil.; 3 Universidade Federal de Juiz de Fora, Faculdade de Enfermagem, Juiz de Fora, MG, Brazil.; 4 Universidade Federal do Espírito Santo, Departamento de Enfermagem, Vitória, ES, Brazil.; 5 Universidade Federal do Rio de Janeiro, Escola de Enfermagem Anna Nery, Departamento de Enfermagem Fundamental, Rio de Janeiro, RJ, Brazil.

**Keywords:** Evaluation Study, Nursing Methodology Research, Nursing Theory, Estudio de Evaluación, Investigación Metodológica en Enfermería, Teoría de la Enfermería, Estudo de Avaliação, Pesquisa Metodológica em Enfermagem, Teoria de Enfermagem

## Abstract

The objective of this study was to evaluate the Theory of Professional Bonds from the description and analysis steps of Meleis’ theory evaluation model. This is a theoretical, analytical, and philosophical study, with the collection of manuscripts through literature review to deepen knowledge regarding the origin, the theoretical and philosophical frameworks, and the practical application of the Theory of Professional Bonds. The study was developed in two steps: procedures for the evaluation using the model proposed by Meleis’ and procedures for validation of the evaluation using the Delphi strategy and the Likert scale. Descriptive-reflective analysis involves an impartial and detailed examination of the theory, and it is possible to define the scope for a middle-range theory based on Leontiev’s Activity Theory. Concepts are defined, delimited and interrelated. It is possible to transcend its applicability from the nursing team to the interdisciplinary team. The theory evaluation advanced by reaffirming the importance of theory to practice and identifying potential for theoretical development, contributing to the elaboration of an unprecedented guideline for theoretical nursing studies.

## RESUMO

O objetivo deste artigo é avaliar a Teoria dos Vínculos Profissionais a partir das etapas de descrição e análise do modelo de avaliação de teorias de Meleis. Trata-se de um estudo teórico, analítico e filosófico, com captação de manuscritos por meio de revisão de literatura para aprofundar os aspectos quanto à origem, aos referenciais teóricos, filosóficos e à aplicação prática da Teoria dos Vínculos Profissionais. O estudo foi desenvolvido em dois momentos: procedimentos para a avaliação por meio do modelo proposto por Meleis e procedimentos para validação da avaliação por meio da estratégia Delphi e escala *Likert.* A análise descritiva-reflexiva envolve um exame imparcial e detalhado da teoria, sendo possível definir o escopo por uma teoria de médio alcance baseada na Teoria da Atividade de Leontiev. Os conceitos são definidos, delimitados e inter-relacionados. É possível transcender sua aplicabilidade da equipe de enfermagem para a equipe interdisciplinar. A avaliação da teoria avançou por reafirmar a importância da teoria para a prática e identificar potencialidades de desenvolvimento teórico, contribuindo para a elaboração de uma diretriz inédita para estudos teóricos de enfermagem.

## RESUMEN

El objetivo de este artículo es evaluar la Teoría de los Vínculos Profesionales desde las etapas de descripción y análisis del modelo de evaluación de las teorías de Meleis. Se trata de un estudio teórico, analítico y filosófico, con la captación de manuscritos mediante una revisión bibliográfica para profundizar en los aspectos relativos al origen, a los referentes teóricos y filosóficos y a la aplicación práctica de la Teoría de los Vínculos Profesionales. El estudio se desarrolló en dos momentos: procedimientos para la evaluación mediante el modelo propuesto por Meleis y procedimientos para la validación de la evaluación según la estrategia Delphi y la escala Likert. El análisis descriptivo-reflexivo implica un examen imparcial y detallado de la teoría y es posible definir el alcance mediante una teoría de rango medio basada en la Teoría de la Actividad de Leontiev. Se definen los conceptos, se delimitan y se interrelacionan. Es posible trascender su aplicabilidad desde el equipo de enfermería al equipo interdisciplinario. La evaluación de la teoría avanzó reafirmando la importancia de la teoría para la práctica, identificando el potencial de desarrollo teórico y contribuyendo para la creación de una directiva sin precedentes para los estudios teóricos de enfermería.

## INTRODUCTION

Nursing theories emerged to constitute a body of knowledge that would meet the interests and peculiarities of the profession, needs of the social context, being a critical element in the development of the nursing discipline^(1,2)^. Thus, over time, they achieved prestige with a type of disciplinary knowledge for guiding practice. However, the particularities of the health area require the application of products and knowledge that can be highly reliably successful, that do not add harm and can have a good cost-benefit ratio. Thus, in the specific case of nursing theories, the evaluation of theories is the main procedure to measure quality and adequacy that guarantee an appreciation of the virtues of “good theory”, both before its comprehensive use and in the steps of refinement by use^(3,4)^.

Almost all nursing theories are guided by clinical phenomena or contexts. However, nursing practice also involves dimensions linked to research, teaching and management. In the management dimension, the Theory of Professional Bonds (TPB), created in 2005, is one of the few theoretical contributions available.

Developed in Brazil, the TPB has been used by nurses and students as a theoretical framework or model to unveil the interpersonal relationships of teamwork in order to build healthy bonds and links^(5)^. Its central proposal is to revitalize the subjective issues surrounding the work process and constitute a nursing management tool, configuring itself as a model for teamwork that observes and values the particular characteristics of the nursing professionals.

Although there are published studies on the results of TPB use, there are no publications that evaluate the theory with the systematic application of an evaluation method. The authors of this article find advantages in carrying out this systematic evaluation, considering that it can determine theoretical adequacy for research, teaching, administration and nursing practice. In addition, different explanations about the same nursing phenomenon can be compared and verified, helping to identify an epistemological approach based on knowledge and contextual and sociocultural understanding of theory theorists^(2)^.

The objective of this study is to evaluate the Theory of Professional Bonds from the description and analysis steps of Meleis’ theory evaluation model.

## METHOD

This is a theoretical study of theory evaluation conducted in two phases: procedures for the evaluation of TPB and procedures for the validation of the results obtained in the first phase.

The authors selected the theory’s internal validation^(4)^, considering that it is a meta-theoretical evaluation of the intrinsic elements to determine the adequacy of its use and the epistemological approach^(3,6)^. For this purpose, the Meleis’ theory evaluation model was chosen because it is philosophically based on a historical view of science, and performs a comprehensive evaluation including description, analysis, criticism, testing and support^(2)^. For this study, the evaluation incorporated theoretical description and analysis, as the test and support refer to aspects of external or empirical theoretical validity^(4)^.

### Phase 1: Procedures for the Theory Evaluation

In this phase, a narrative literature review was carried out with the purpose of identifying the published manuscripts about TPB that served as sources for the theoretical analysis. For the search, the following descriptors were used: Theory, Professional Bonds, Healthy Environments and Interpersonal Relationships. The review included published materials on TPB with conceptual, theoretical and methodological approaches published between 2005 and 2017, coming from electronic environments and gray literature, including the original manuscript of the theory and other texts by its authors.

The evaluation of TPB was performed by the main researcher, using Meleis’ theory evaluation model, which includes five steps: description, analysis, criticism, testing, support. Each step, in turn, has its criteria and respective evaluation units^(2)^. For this study, what was recommended in the first two steps of the evaluation model was followed: description and analysis.

The choice of the description and analysis steps is in line with the recommendation to recognize and identify the limits for the theoretical evaluation task, considering the degree of exposure to the theory and the time spent to understand the theory. From the general appreciation of the narrative review material, the researchers did not find studies that performed the steps of TPB description or analysis. These steps indicate the main elements of theory construction. They also judged that the time taken to evaluate the five steps would make it difficult for the experts to engage in the second step, when the Delphi technique was used, which could impact its quality. Finally, the technique with experts used in the second step was original, which would require a severe effort of coordination and possible adjustments, being more desirable to limit the metatheoretical task to fewer steps. Thus, it was understood that criticism, testing and support could be carried out in the future due to its property closer to use, refinement and theoretical updating.

**Description step:** systematic reading of the theory to obtain a vision of the author’s work, the objective, the questions to be asked, as well as the answers to be found. The description step comprises two types of components: the structural ones, having assumptions, concepts and propositions as units of analysis, and the functional ones, in which the focus, client, nursing, health, environment, nurse-client interaction, nursing problems and nursing therapy are analyzed^(2)^.

**Analysis step:** content examination to identify theory components. Useful for development and examination, with sub-steps of concept analysis and theory analysis^(6)^.

**Concept analysis sub-step:** comprises the criterion of differentiation from others through the following units of analysis: semantic definition, logical and contextual derivation; background; consequents, and examples^(2,6)^.

**Theories analysis sub-step:** allows understanding the questions of the theory, the phenomena and the strategies that are important for its development, all this considering the influencing factors in the development of the theory until its current configuration. Theoretical, paradigmatic origin and internal dimensions are criteria^(2,6)^.

The product of the description and analysis was a descriptive- reflective writing type report, considered the most appropriate by the research team. Meleis’ evaluation model is not exhaustive about the types of studies to be produced, accepting formats such as interpretive analysis, theoretical-reflective study, descriptive-reflective study or literature review^(6)^.

### Phase 2: Procedures for Validation

The second moment aimed to validate the results of the first phase. For evaluation, a panel of experts was set up guided by the Delphi consensus technique^(4)^.

The recruitment of experts used criteria of scientific production, professional experience in the area of theoretical conceptions in Nursing or with TPB^(4)^. Data for preliminary selection were obtained from the Lattes Platform. Recruitment, by invitation, included the communication of study justifications, objectives, expected impacts, form of evaluation and deadline for response. After accepting and signing the Informed Consent Form, the participants entered the data collection phase for validation.

Data collection from the Delphi Panel took place via email. Initially, a manuscript containing the criteria for evaluating TPB was sent to panelists, based on the parameters of Meleis’ theory evaluation model. Subsequently, data were collected to characterize the experts and obtain essential evaluation elements for the consensus judgment.

The manuscript for consensus included the items of the theoretical evaluation carried out by the main researcher, in the content of description and analysis steps. The description items are assumptions, concepts, propositions, focus, client, nursing, health, nurse-client interaction, environment, nursing problems and nursing therapeutics. In the analysis, the following items stand out: differentiation from others, characteristics of the theorist, paradigmatic origin and internal dimensions.

Items were judged using a five-point Likert scale (totally agree, partially agree, neither agree nor disagree, partially disagree, totally disagree) to measure the individual agreement of each expert with the assessment performed. A percentage of ≥80% agreement with a cut-off point was used for consensus.

### Ethical Aspects

All ethical and legal requirements for research with human beings were met, with approval by the Research Ethics Committee, with the certificate of research approval number 3237583 of 2019.

## RESULTS

**Chart 1.**
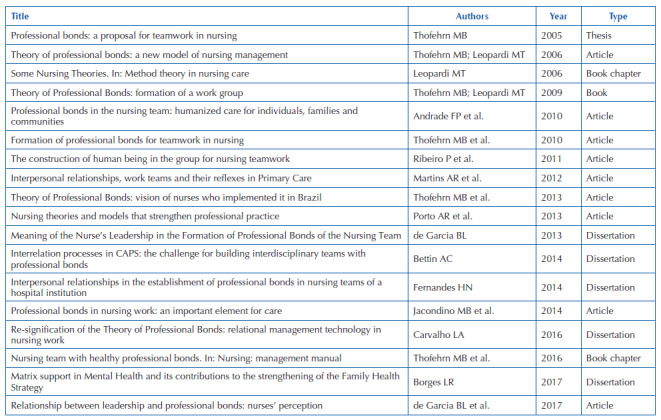
Summary of characteristics of included studies – Juiz de Fora, MG, Brazil, 2022.

The bibliographic review made it possible to identify 18 studies, which are summarized in Chart 1.

For validation, three judges who met the criteria of expertise participated, among the five invited to participate. The participants are women, aged between 29 and 54 years old, belonging to the South and Southeast regions of Brazil, nurses, being 2 doctoral students with a master’s degree in nursing and a doctor in nursing. All had experience in nursing theories, ranging from three to nine years. One of the evaluators did not know the TPB, while the others had knowledge and experience with the theory, 12 and 7 years.

In the first round, a consensus was not reached for the evaluation units of nursing problems and nursing therapeutics in the description step, which obtained an agreement rate of 66.7% and 73.3%, respectively. However, the assumptions, concepts, propositions, focus, client, nursing, health, nurse-client interaction and environment units obtained 93.3% agreement.

Furthermore, among the analysis components, differentiation from other concepts and internal dimensions did not reach the minimum rate of 80%, with an agreement rate of 73.3% each. All other theoretical and paradigmatic evaluation units reached an agreement of 93.3% each.

Only those who did not obtain an agreement in the first round were re-evaluated in the second round. In the new evaluation, agreement rates were: 93.3% for “nursing problems” and “nursing therapy” units and 100% for the analysis of “differentiation of other concepts” and “internal dimensions”. Table 1 and Table 2 summarize the theoretical evaluation.

**Table 1.** Synthetic results of the description step – Juiz de Fora, MG, Brazil, 2022.

Criteria	Evaluation unit	Results according to consensus
**Structural components**	**Assumption**	Assumptions built under the constructivist philosophical viewpoint, in addition to considering the Marxist critique
	**Concepts**	The concepts were present, being visible and apparent, relating the thought of the theorists and relating to each other. They are presented in an organized, logical, and articulated manner. They have a high level of abstraction
	**Propositions**	Propositions still require further development
**Functional components**	**Focus**	Dialogical interaction between interpersonal relationships and subjectivity
	**Client**	The nursing team needs encouragement to understand subjectivity
	**Nursing**	It is strengthened as a discipline and profession, with a focus on therapeutic care
	**Health**	It considers the biological, psychological, social and spiritual dimensions of the worker
	**Nurse-client interaction**	Related to professional bonds formed by the nursing teamwork to achieve therapeutic care
	**Environment**	Built from the relief of suffering present in normative activities through the formation and affirmation of healthy professional bonds
	**Nursing problem**	It is the difficulty for human beings to understand their subjectivity
	**Nursing therapeutics**	The mediating tool is the model for nursing teamwork

**Table 2.** Synthetic results of the analysis step – Juiz de Fora, MG, Brazil, 2022.

Step	Criteria	Evaluation unit	Results according to consensus
**Concept analysis**	**Differentiation from others**	Definitions (semantics, logic, contextual), Antecedents, Consequences, Examples	Central concept: professional bonds. It is an abstract expression that is vital for mental and experimental formulations in theory building. It is a dynamic structure that projects shared ways of conducting work. The bonds can have marks of expropriation or strengthening of subjectivity
**Theory analysis**	**The theoretical**	Background education, Practical experience, Professional Network, Sociocultural Context	Through their professional and pedagogical careers, the theorists consolidated the TPB. Due to the regional context of the authors and the concentration of studies in the region of professional experience, it is suggested that the TPB be applied to other social and geographical contexts in addition to those already included for verification of external (empirical) validation
	**Paradigmatic origins**	References, Citations, Assumptions, Concepts, Propositions, Hypotheses, Laws	The paradigm of historical-dialectical materialism guided the construction of the TPB
	**Internal dimensions**	Justification/logic that the theory was built, system of relationships, content, beginning of theory, scope, goal, context, abstraction and method	After the evaluation, TPB is classified by the level of abstraction as a middle-range theory; since it refers to a specific phenomenon, it has a more limited scope, but it has a certain degree of abstraction

## DISCUSSION

Evaluation of theories with systematic criteria is found in nursing theory books developed by one or a few meta-theorists, without peer judgment of the material produced. Although this evaluation by an experienced meta-theorist is valuable, the incorporation of judges, the application of Delphi and the use of agreement criteria represent elements that can add more reliability to an internal validation of the theory^(4)^.

The study results indicate the successful realization of the Delphi of systematic theoretical evaluation with the Meleis’ model^(2)^. This is confirmed when considering the high values of agreement for most items since the first round, as well as the permanence of the judges in all steps of the process. It is understood that the design with four evaluators was in line with the characteristic of the consensus Delphi, since the high levels of expertise in TPB of the judges justify the composition of teams with fewer components^(4)^.

## DESCRIPTION OF THE THEORY OF PROFESSIONAL BONDS

### Structural Components

TPB is based on assumptions articulated with the work process, human activity and group dynamics. The research that originated the theory was influenced by society’s values, which reflects the congruence with Vygotsky, especially the values related to theorists, since subjectivity and the sociocultural context in which the theories are inserted are always considered in the theory.

As a priori truth, the assumptions are implicit in the TPB, indicating its sociological affiliation. For example, TPB understands the work process from a Marxist perspective, perceiving it as a relational movement of the nursing team. It presupposes that the work itself is the professional task, that is, the therapeutic care that aims to transform the state of pain and suffering into a state with more comfort. On the other hand, the object of work consists of the biological body of the human being, which is also a consumer of this work. Theorists consider these assumptions as having identification with constructivist philosophy and consider the Marxist critique. However, in the theoretical evaluation, it was only possible to perceive this perspective through the set of previous knowledge of the authors, their beliefs and their values.

The propositions mainly refer articulating the concepts of bonding with human and work relations, mediated by the actions of the nurse and coordinator of the work group. Regarding the TPB propositions, it is highlighted that there is still a challenge in understanding what would be the propositions in theory. Meleis, for example, when mentioning the term propositions, refers to two types – existence propositions and relational propositions^(2)^ –, although, in fact, existence and relational refer to statements and not to propositions^(7,8)^. It is not difficult to suppose that these metatheoretical overlaps in the use of the terms affirmatives and propositions make proposition construction a challenge for theorists and possibly an object of confusion.

The content of the theory is articulated through more abstract concepts and relational propositions, so the core concept is professional bonds, derived from Pichon-Rivière’s bonds theory, in addition to some formulations of the work process of Karl Marx and ideas from Leontiev’s Activity Theory^(5)^. The representativeness of the concept of professional bonds in TPB is directed to the importance of approaching group relations, to facilitate the development of activities through the feeling of belonging of the team members.

In addition to the concept of professional bonds, TPB is supported by other concepts, such as: professional task, work object, work instrument, workforce, purpose or product of work, dynamics of a work group, action and discourse, development of a group, task of a group, rules, community, and division of labor.

### Functional Components

The focus of TPB is centered on two phenomena: interpersonal relationships and issues of the subjectivity of the nursing teamwork. TPB is concerned with the experience of a group gathered for a specific action, in which the main character is to deal with human relationships and, concomitantly, with the dynamics of work relationships. In addition, the TPB does not only present the assigned concepts, but also deals with how to operationalize them through the teamwork model.

So, the nursing team is the client for TPB, as it requires encouragement and guidance from the nurse, leader and coordinator, to clarify subjectivity. Thus, nurses must initially access their subjectivity and understand themselves, and then identify and understand people’s individual characteristics.

Theorists consider nursing as an ancient activity of caring for people and constituting itself as a professional discipline that must be understood from its work process. Commonly, the phenomena that involve the sick person and the care for them are the emphasis. Care of team members individually or as a whole, relevant to TPB as a theory for the professional caregiver, is often not the focus. Its theoretical components are oriented to team members’ phenomena, with the goal of nursing strengthened as a profession and discipline, without losing focus on therapeutic care.

Theorists understand that nurses, when appropriating the concepts of TPB, would be using tools to identify and manage relational situations inherent to the work group. The construction of this interaction between professionals considers the biological, psychological, social and spiritual dimensions of the worker. Therefore, health is achieved in the environment of relationships, in conditions of interaction built from the relief of suffering present in normative and routine activities^(9)^. It is a theory for nurses.

The concept of environment, derived from Vygotsky’s triangular scheme and expanded by Leontiev, is represented in the socio-historical context. In it, every activity is inserted to include individual internal issues of team members or internal to the group, as well as issues external to the group^(5)^. This environment also has its nursing problems, incorporating issues related to the difficulty that team members have in accessing and recognizing their subjectivity and that of the other, extrapolating misunderstandings and communication failures.

The nursing practice environment is a complex construct, investigated mainly in the United States of America, with theoretical foundations grounded in the sociology of organizations, occupations and work^(10)^. This construct of practice environment is different from the user’s or client’s environment concept, recurrent in nursing theories, and is one of the concepts of the nursing metaparadigm^(11)^. Thus, TPB expands another environmental dimension related much more to a place or working condition built on healthy professional relationships.

For the concept of nursing problems, TPB presents itself as prescriptive, providing a model of teamwork as a nursing therapy. It consists of four steps that guide nurses to involve and lead the nursing team towards the formation and affirmation of healthy professional bonds: definition of goals and purposes, group formation, group development and group closure.

In the TPB evaluation, the nurse-client interaction criteria can only be judged indirectly, given the centrality of professional bonds, or in a triarchic dimension such as “health professional- health professional-client”. This perspective can be added to the most familiar domain of nursing theories, which is that of client-nurse^(12)^.

## ANALYSIS OF THE THEORY OF PROFESSIONAL BONDS

### Concept Analysis

The core concept evidenced in the TPB corresponds to professional bonds, which is an abstract term, with a conceptual meaning of a fundamental approach to mental and experimental formulations in the construction of theory. From a philosophical point of view, “professional bonds” represents a theoretical term, that is, its meaning depends on the theory to which it relates^(13)^. Because of this, the use of any term linked to direct or indirect observables of the theoretical term professional bonds should use TPB as a conceptual framework of reference, mainly based on the definition presented. The concept of professional bonds, acting as a theoretical term, is defined as “the configuration of interpersonal relationships in small work groups”. They can be bonds with marks of expropriation or marks of strengthening of subjectivities.

The TPB evaluation showed semantic clarity, that is, when the constitutive definition for the concept is clear and very distinguishable in content. Furthermore, semantic coherence is verified by the absence of conflicts in the use of different definitions, considering stability and coherence with the constitutive definition used for the concept.

The core concept of professional bond has dimensions and components such as interpersonal relationships, group dynamics and subjectivity. They are interrelated to present a single dimension for nursing practice, without contradictions in relational propositions, which indicates the fulfillment of the structural consistency criterion.

On the other hand, the concept of healthy bonds is, in many ways, logical. Although intuitively easy to understand, readers may confuse it with employment relationships, such as public or private workers^(14)^. The meaning of healthy bonds in TPB has a logic constituted by daily professional experiences, even when considering empirical findings. In addition, the imaginary, the symbol, anxieties and desires are considered in the construction of interpersonal relationships developing of the nursing team’s activities^(9,15)^.

Again, the idea of a theoretical term and its relationship with the conceptual system of the theory is used^(13)^. For some authors, connecting a concept, construct or a theoretical term to the entire conceptual system and languages of a particular theory could prevent its inappropriate use^(16)^. Considering this perspective, concepts are not theory building blocks, but elements that must be “understood in perspective”, from the theory that uses them. Therefore, the professional bond gains meaning in the theory that supports it.

### Theory Analysis

One of the importance of theory analysis can be to confront the unacceptability of a theory by clarifying the structuring aspects of theorists and theory^(2)^. In the case of TPB, the analysis mainly fits the criterion of improvement and theory development.

Due to the professional trajectory of care, teaching and research of both theorists, it is noticeable that the theory was developed from knowledge derived from other courses. It converges to the philosophical current or paradigm of dialectical historical materialism, compatible with the temporal context of the theory and its references.

The rationale of TPB refers to a theory concatenated in structure as it is built from related concepts. However, some need improvements, such as rules, division of labor and community and the other included, such as spirituality.

TPB was built by a system of relationships between phenomena to explain the elements. Thus, the construction of the theory used the field method in which the phenomena are explained by their relationships. The relationship phenomenon is the professional bonds that can be influenced by elements such as the sociocultural context and subjectivity.

Concerning the level of abstraction, TPB can be characterized as a middle-range theory^(2)^, as it relates to a specific phenomenon of daily nursing practice, has a more limited scope and still has a certain degree of abstraction.

Despite the authors idealizing and developing a conceptual structure based on other theorists, it was through a particular survey of concrete cases of reality that they observed it, with the construction of premises through saturation, to reveal the phenomenon. When relating phenomena and concepts to each other, theorists consolidate the generalization or the inductive process, starting from the particular to the generalization.

Regarding the purposes, TPB presents structural components of a prescriptive theory for defining the client’s situation – nursing therapy through the nursing teamwork model –, which configures the process by which the therapy is implemented, signaling the patterns of desired responses, as well as the affirmation and formation of healthy groups.

Despite the empirical validation of the theory with nursing teams, it was possible to identify that the TPB has advanced in applicability to interdisciplinary teams^(9,15,16)^. However, it is limited as it does not present a generalized test, proven by the lack of studies in other countries. All studies are concentrated in the southern region of Brazil.

## IMPLICATIONS OF EVALUATION OF TPB FOR USE AND DEVELOPMENT

Maira Buss Thofehrn created the TPB in partnership with her advisor Maria Tereza Leopardi in the early 2000s. Even today, the theory continues to be used as a reference to redefine the micro space of work^(16,17)^, investigate interpersonal relationships and the work process^(15,18)^, among other applications in research. However, the metatheoretical development of TPB can bring new perspectives for its use in professional practice.

Supposedly, a reorganization of elements of the TPB as a Professional Practice Model (PPM) can extend its empirical foundation in the middle range. A PPM is, by nature, a theoretical framework that describes how nurses carry out their practice, collaborate, communicate and develop professionally^(19,20)^. PPMs basically aggregate in their structure organizational concepts and concepts of interest to nursing, core corporate values and an external or nursing or organizational theory of reference. Its recurring components are the following elements: patient outcomes, leadership, independent practice, collaborative practice, environment, development and recognition, research and innovation^(21)^. The TPB has interchangeable components with the structure of the PPM, such as the centrality in the creation of professional bonds, the emphasis on the human being, the formation of a flexible and adaptable team, the expanded use of the concept of community, involving people, health services and the socio-historical context, as well as the modeling of work with cooperation and procedural integration, an environment for the relief of suffering, and the formation and affirmation of healthy professional bonds.

## FINAL CONSIDERATIONS

The study allowed the evaluation of TPB from the description and analysis steps of Meleis’ theory evaluation model, highlighting its descriptive, analytical and epistemological aspects. Thus, it makes it possible to clarify the understanding of the theory, professional bonds and the teamwork model, advancing knowledge about nursing theories.

The interpretation of the results indicated that TPB is a middle-range theory, having the core concept of professional bonds and other concepts at a high level of abstraction, guided by the paradigm of historical-dialectical materialism, with constructivist and Marxist assumptions. In addition, it incorporates a description for the four concepts of the nursing metaparadigm, with a focus and problem on the emphasis of relationships and subjectivities, indicating that the theory was built from the professional careers of the theorists.

This evaluation of the TPB may facilitate the understanding of nursing students and professionals about the theory and encourage its application in care. On the other hand, it also reinforces the importance of caring for those who care, by emphasizing subjectivity and interpersonal relationships, valuing the nursing team’s professional.

Other theory evaluation studies using different systematized methods are suggested. With the expansion of the use of theory, an evaluation with the other phases of the Meleis’ theory evaluation model is recommended to judge elements that still depend on a broad application of the TPB.

## ASSOCIATE EDITOR

Marcia Regina Cubas
